# IMA genome‑F17: Draft genome sequences of an *Armillaria* species from Zimbabwe, *Ceratocystis colombiana*, *Elsinoë necatrix*, *Rosellinia necatrix*, two genomes of *Sclerotinia minor*, short‑read genome assemblies and annotations of four *Pyrenophora teres* isolates from barley grass, and a long-read genome assembly of *Cercospora zeina*

**DOI:** 10.1186/s43008-022-00104-3

**Published:** 2022-11-21

**Authors:** Brenda D. Wingfield, Dave K. Berger, Martin P. A. Coetzee, Tuan A. Duong, Anke Martin, Nam Q. Pham, Noelani van den Berg, P. Markus Wilken, Kiruba Shankari Arun-Chinnappa, Irene Barnes, Sikelela Buthelezi, Buddhika Amarasinghe Dahanayaka, Alvaro Durán, Juanita Engelbrecht, Alice Feurtey, Arista Fourie, Gerda Fourie, Jesse Hartley, Eugene N. K. Kabwe, Mkhululi Maphosa, Deborah L. Narh Mensah, David L. Nsibo, Lizel Potgieter, Barsha Poudel, Eva H. Stukenbrock, Chanel Thomas, Niloofar Vaghefi, Tanya Welgemoed, Michael J. Wingfield

**Affiliations:** 1 Department of Biochemistry, Genetics and Microbiology, Faculty of Natural and Agricultural Sciences, Forestry and Agricultural Biotechnology Institute (FABI), University of Pretoria, Pretoria, South Africa; 2 Department of Plant and Soil Sciences, Forestry and Agricultural Biotechnology Institute (FABI), University of Pretoria, Pretoria, 0028 South Africa; 3 Centre for Crop Health, University of Southern Queensland, Toowoomba, QLD 4350 Australia; 4 PerkinElmer Pty Ltd., Level 2, Building 5, Brandon Business Park, 530‑540, Springvale Road, Glen Waverley, VIC 3150 Australia; 5 Plant Health Program, Research and Development, Asia Pacific Resources International Holdings Ltd. (APRIL), Pangkalan Kerinci, Riau 28300 Indonesia; 6 Environmental Genomics, Max Planck Institute for Evolutionary Biology, 24306 Plön, Germany; 7 Environmental Genomics, Christian-Albrechts University of Kiel, 24118 Kiel, Germany; 8 Department of Biochemistry, Genetics and Microbiology, Centre for Bioinformatics and Computational Biology, Forestry and Agricultural Biotechnology Institute (FABI), University of Pretoria, Pretoria, South Africa; 9 School of Agriculture and Food, University of Melbourne, Parkville, VIC 3010 Australia; 10 CSIR, Food Research Institute, Accra, Ghana

## IMA GENOME‑F 17A

### Draft genome sequence of an *Armillaria* species from Zimbabwe

#### Introduction

The genus Armillaria includes at least 38 species, most of which are facultative necrotrophs (Gregory and Rishbeth 1991). Pathogenicity of these organisms can result in *Armillaria* root and stem rot and what is referred to as shoestring root rot (Morrison 1991). This disease can bring about massive devastation to woody plants grown for horticulture, agriculture, as well as natural and managed forests across the various continents (Baumgartner and Rizzo 2001, 2002; Guillaumin et al. 1993; Labbé et al. 2015). The saprophytic nature of some *Armillaria* spp. results in enhancement of forest ecosystems through the breakdown of woody material, resulting in carbon and mineral cycling (Baumgartner et al. 2011; Heinzelmann et al. 2019). Transition from a saprophytic to a pathogenic lifestyle, and vice versa, can occur due to intra-species variation, forest management systems, the state of the host (e.g. stressed or healthy), as well as environmental factors (e.g. elevation) (Legrand et al. 1996; Prospero et al. 2004; Tsykun et al. 2012).

Various groups have conducted omics-based research on *Armillaria* species (Akulova et al. 2020; Anderson et al. 2018; Caballero et al. 2022; Collins et al. 2017; Collins et al. 2013; Heinzelmann et al. 2020; Kolesnikova et al. 2019; Linnakoski et al. 2021; Misiek et al. 2011; Misiek and Hoffmeister 2012; Sipos et al. 2017; Sonnenbichler et al. 1997; Sun et al. 2020; Wingfield et al. 2016a, b; Zhan et al. 2020). These genomics, proteomics and metabolomics studies were done to gain insight into the molecular mechanisms and biochemical properties that drive the pathogenicity and virulence of *Armillaria* spp. This information would eventually help to develop efficient strategies for identifying these fungi, containing their spread, and minimising damage to forest ecosystems.

The previously determined nuclear and mitochondrial genomes of various *Armillaria* species are providing invaluable resources for genome-based research (Table 1). Studies using these genomes have broadened our understanding of the biology of the *Armillaria* species and the evolution of their genomes. For example, Sipos et al. (2017) showed that genome evolution in the genus was predominantly caused by gene family expansion. Kolesnikova et al. (2019) assembled the complete mitochondrial genomes of *A. borealis, A. gallica, A. sinapina,* and *A. solidipes* and found a high degree of variation in size, gene content and genomic organization among these phylogenetically closely related species. Recently, the first chromosome-level *Armillaria* genome assembly became available, revealing genome-wide recombination in the genome of *A. ostoyae* (Heinzelmann et al. 2020).

Sequenced *Armillaria* species originate primarily in the Northern Hemisphere. The genome of only one species from the Southern Hemisphere, *A. fuscipes* from South Africa, has so far been published (Wingfield et al. 2016a, b). It is known that species from the Northern Hemisphere, Australasia together with Southern America, and Africa, respectively, reside in distinct monophyletic lineages (Coetzee et al. 2011; Koch et al. 2017). Genomes of species in these geographic locations may, therefore, have followed very different evolutionary pathways. Within the African clade, Coetzee et al. (2005) identified two lineages, referred to as *A. fuscipes* and African Group B. Here, we report the genome of an *Armillaria* isolate belonging to African Group B (sensu Coetzee et al. 2005), sequenced using both long- and short-read technologies. This genome expands the sequence resources for *Armillaria* species from the Southern Hemisphere and Africa.

#### Sequenced strains

**Zimbabwe:** Stapelford, Manicaland isolated from *Brachystegia utilis,* 2001, *E. Mwenje* (culture CMW4456; PREM 63337—dried culture).

#### Nucleotide sequence accession number

The Whole Genome Shotgun project of the *Armillaria* sp. genome has been deposited at DDBJ/ENA/GenBank under the accession JANDKJ000000000. The version described in this paper is version JANDKJ010000000.

#### Materials and methods

The culture of isolate CMW4456 were grown and maintained in MYA (1.5% Malt extract, 0.2% Yeast extract, 1.5% Agar) at 24 °C in the dark for 4 weeks. DNA was extracted from the harvested mycelia using the method described by Duong et al. (2013). PacBio sequencing was conducted on the Sequel IIe system using the circular consensus sequencing (CCS) mode at Inqaba Biotechnical Industries (Pty) Ltd. (Pretoria, South Africa).

For short read sequencing on the Illumina HiSeq platform, genomic DNA was extracted from cultures grown in MY broth (1.5% Malt extract, 0.2% Yeast extract) for six weeks at 24 °C in the dark. Harvested cultures were kept at − 80 °C, followed by lyophilization. DNA was extracted with the Qiagen DNEasy Plant Pro Kit (50) Cat. No. 69204 (Qiagen, Sandton, South Africa) following the manufacturer’s instructions. Illumina paired-end library preparation and whole-genome sequencing was done with an insert size of 350 bp and read-length of 150 bp at Macrogen.

Trimmomatic v. 0.38 (Bolger et al. 2014) was used to trim adapter sequences and low-quality ends of the Illumina reads (ILLUMINACLIP, TruSeq3-PE.fa:2:30:10:8; LEADING, 3; TRAILING, 3; MINLEN, 30).

The PacBio HiFi reads were assembled with CLC Genomics Workbench v 22.0.1 (QIAGEN, Aarhus). The assembly was subsequently polished with the trimmed Illumina HiSeq reads, using Pilon v. 1.23 (Walker et al. 2014). Genome completeness was evaluated with Benchmarking Universal Single-Copy Orthologs (BUSCO) v. 5.3.2, using the agaricales_odb10 lineage dataset (Manni et al. 2021). AUGUSTUS v. 3.4.0 (Keller et al. 2011; Stanke et al. 2008; Stanke and Waack 2003) was used to predict protein coding genes, applying gene models of the closely related species, *Coprinus cinereus*. QUAST v 5.0.2 (Gurevich et al. 2013) was used to evaluate metrics, including contig number, total length, GC content, and N50 for the genome assemblies. BUSCO, AUGUSTUS and QUAST were run using the Galaxy platform (Afgan et al. 2018; The Galaxy Community 2022) (https://usegalaxy.eu/). *Armillaria ostoyae* strain C18/9 genome (Sipos et al. 2017) with accession number FUEG00000000.1 was used as the reference genome for genome quality evaluation.

The identity of the *Armillaria* African Clade B isolate CMW4456 for which a genome was sequenced was confirmed based on phylogenetic grouping with published DNA sequences. DNA sequences from the internal transcribed spacer region (ITS) and the translation elongation factor one alpha (tef1-α) were extracted from the genome. Since few tef1-α sequences from African isolates are available in databases, the tef1-α sequence was compared to sequences on GenBank using BLASTn. The ITS sequence was included in the data matrix of Coetzee et al. (2005) and aligned using the online version of MAFFT v. 7. (Katoh et al. 2019). The TrN + G nucleotide substitution model was determined as best fitting the sequence alignment, using jModelTest and the Akaike Information Criterion (Darriba et al. 2012; Guindon and Gascuel 2003), and incorporated in the maximum likelihood analyses. A maximum likelihood phylogenetic tree was constructed using PHYML v. 3.0 (Guindon et al. 2010), applying 1000 bootstrap replicates. The tree was rooted with sequences of *A. hinnulea*.

#### Results and discussion

The 4 PacBio read length ranged between 289 and 15627 bases. The 2 × 151 bp Illumina HiSeq paired-end libraries yielded a total of 14,927,540,182 reads, amounting to 98,857,882 nucleotides. The PacBio and Illumina reads were assembled into 840 contigs with a total assembly size of 54.95 Mbp. All contigs were longer than 1000 bp, with the largest contig being 1,463,441 bp. The N50 and N75 values were 128,967 bp and 45,059 bp, respectively. The L50 and L75 values were 85 and 270 contigs, respectively. The GC content of the assembled genome was 46.53%. Genome completeness was estimated to be 98%, corresponding to 96.8% complete and single-copy BUSCOs, 1.2% complete and duplicated BUSCOs, 0.1% fragmented BUSCOs, and 1.9% missing BUSCOs (n = 3870). AUGUSTUS predicted 13,600 protein coding genes.

The genome statistics of the sequenced *Armillaria* strain correlated with that reported for the genomes of other species in the genus (Table 1). The assembly size fell within the range of 53.00–73.74 Mbp, though the number of predicted protein coding genes (13,600 genes) was somewhat lower than the 14,473–26,261 genes reported in the assembled genomes of other species of *Armillaria.* The GC contents of the genomes of other *Armillaria* spp. (47.4–49.1%) are similar to the GC content of 46.53% reported here.

**Fig. 1:**
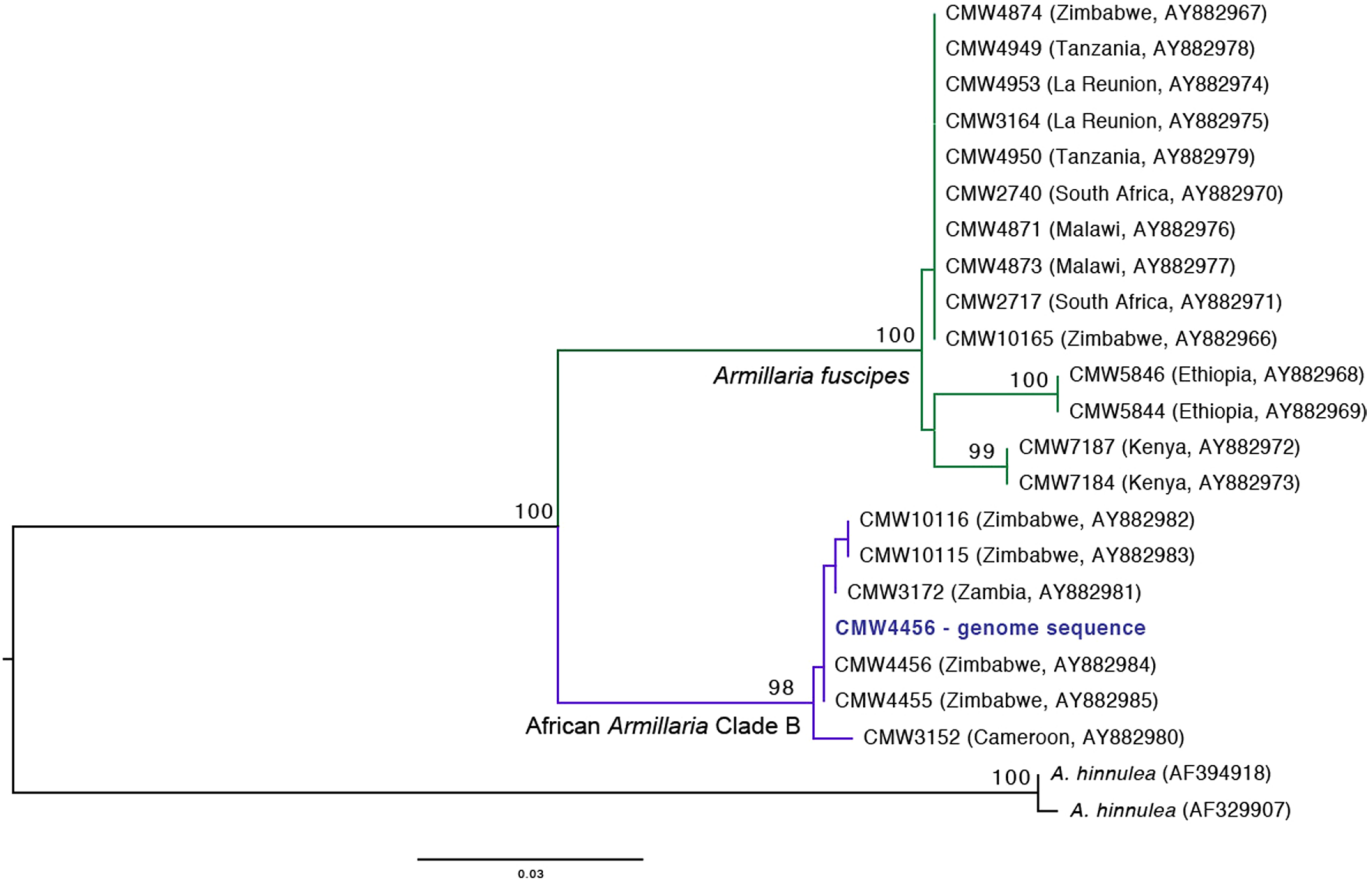
Maximum likelihood tree based on ITS sequence data, confirming the identity of the *Armillaria* African Clade B sp. strain CMW4456 sequenced in this study (highlighted in bold). Bootstrap values above 80% are shown above the nodes. The scale bar represents nucleotide substitutions per site

The sequenced genome of *Armillaria* sp. strain CMW4456 grouped with other strains of the African *Armillaria* Clade B from Cameroon, Zambia and Zimbabwe (Coetzee et al. 2005), confirming its identity (Fig. 1). The tef1-α sequence from the genome was identical to the tef1-α sequence of CMW4456 on GenBank (accession number DQ435617.1). This genome sequence will serve as a useful resource for investigating the biology, chemistry, and pathogenicity of *Armillaria* species from Africa in comparison to those from other continents.

**Authors:** Deborah L. Narh Mensah^1^, Brenda D. Wingfield^1^, Mkhululi Maphosa^1^, Tuan A. Duong^1^, and Martin P. A. Coetzee^1,*^.

^*^Contact: martin.coetzee@fabi.up.ac.za.

**Table 1:** Genome information for the published *Armillaria* species in comparison to *Armillaria* African Clade B isolate CMW4456

Species	Strain	Number of scaffolds	Assembly size (Mbp)	Number of predicted protein coding genes	GC content (%)	Origin	References
*Armillaria altimontana* (NABS X)	837–10	100	73.74	19,326	47.8	USA	Caballero et al. (2022)
*Armillaria borealis*	AB13-TR4-IP16	44,365	66.59	21,969	N/A	Russia	Akulova et al. (2020)
*Armillaria cepistipes*	B5	182	75.52	23,461	47.6	Italy	Sipos et al. (2017)
*Armillaria fuscipes*	CMW2740	24,403	52.98	14,515	N/A	South Africa	Wingfield et al. (2016a, b)
*Armillaria gallica*	Ar21-2	319	85.34	25,704	47.5	USA	Sipos et al. (2017)
*Armillaria gallica*	012 m	63	87.31	26,261	47.4	China	Zhan et al. (2020)
*Armillaria mellea*	DSM 3731	4,377	58.36	14,473	49.1	France	Collins et al. (2013)
*Armillaria ostoyae*	C18/9	106	60.11	22,705	48.3	Switzerland	Sipos et al. (2017)
*Armillaria solidipes*	C28-4	229	58.01	20,811	48.4	USA	Sipos et al. (2017)
*Armillaria solidipes* (form *A. ostoyae*)	ID001	72	55.74	16,357	48.3	USA	Caballero et al. (2022)
*Armillaria* African Clade B sp.	CMW4456	840	54.95	13,600	46.5	Zimbabwe	Described here

*N/A* Not available

## IMA GENOME‑F 17B

### Draft genome sequence of *Ceratocystis colombiana*

#### Introduction

The ascomycete fungal genus *Ceratocystis* consists of over 40 species, many of which are important pathogens of forestry and agricultural tree crops worldwide (de Beer et al. 2014). While many species of *Ceratocystis* only cause problems in their regional distribution, some managed to spread across continents and pose considerable threats to the health of forestry and plantations world-wide (Engelbrecht et al. 2004; Liu et al. 2021).The most notable pathogens of the genus include *C. fimbriata* that causes black root of sweet potatoes (Halsted 1890), *C. platani* that causes cankers and wilt disease of plane trees (Tsopelas et al. 2017), *C. eucalypticola* that causes vascular wilt in *Eucalyptus* (Roux et al. 2020), and *C. manginecans* that causes vascular wilt disease in Acacia and Mango trees (Tarigan et al. 2011; Al Adawi et al. 2013). It also includes the recently described *C. huliohia* and *C. lukuohia* associated with the rapid death of native ˋōhiˋa lehua (*Metrosideros polymorpha*) in Hawai`i (Barnes et al. 2018).

The genomic resources for studying *Ceratocystis* species have been increasing in recent years thanks to the increasing affordability in sequencing fungal genomes with next generation sequencing technologies. There are a number of genome sequences available for species of *Ceratocystis* from various hosts including *C. fimbriata* from sweet potato (Wilken et al. 2013), *C. manginecans* from *Acacia mangium* (van der Nest et al. 2014b), *C. eucalypticola* from *Eucalyptus* (Wingfield et al. 2015a, b), *C. albifundus* from *Acacia mearnsii* (van der Nest et al. 2014a, 2019), *C. harringtonii* from poplar (Wingfield et al. 2016a, b), *C. smalleyi* from hickory (Wingfield et al. 2018), and *C. cacaofunesta* from cacao (Molano et al. 2018). In this study, we report the genome sequence of an isolate of *C. colombiana* from *Coffea arabica* in Colombia. This species is known from coffee, citrus, and *Schizolobium parahyba* and but so far is only known from Colombia (Van Wyk et al. 2010). Pathogenicity assays showed that that *C. colombiana* can cause disease on *Coffea arabica* and hence it could pose a serious threat to the coffee industry in the region and the availability of genome sequence data will help in understanding its biology and pathogenicity.

#### Sequenced strain

**Colombia:** Valle del Cauca, isol. ex *Coffea arabica*, 2000, *M. Marin* (CMW5751; CBS 121792; PREM 59434—dried culture).

#### Nucleotide sequence accession number

The *Ceratocystis colombiana* genomic sequence data has been deposited at DDBJ/EMBL/GenBank under the accession JAOSLS000000000. The version described in this paper is version JAOSLS010000000.

#### Materials and methods

A single spore culture of *C. colombiana* CMW5751 was grown on malt extract broth (2% malt extract; 0.5% yeast extract) for 3 d at room temperature, after which the mycelium was harvested and freeze-dried. DNA was extracted from freeze-dried mycelium using the method described in Duong et al. (2013). Whole genome sequencing was by Macrogen (Seoul, South Korea) where a paired-end library was constructed using the TruSeq PCR free protocol and sequenced on the HiSeq 2500 platform to obtain 251 bp paired-end reads. The illumina data were trimmed using Trimmomatic v.0.38.1 (Bolger et al. 2014) and the genome was assembled using SPAdes v.3.14.0 (Bankevich et al. 2012). The resulting assembled scaffolds were filtered based on k-mer coverage (≥ 20% of the medium coverage) and size (≥ 500 bp). Assembly completeness was assessed using BUSCO v.4.1.4 (Simão et al. 2015) using the sordariomycetes_odb10 dataset. The number of protein coding gene models was estimated using AUGUSTUS v.3.2.3 (Keller et al. 2011) using the pre-defined species model for *Fusarium graminearum*. To validate the identity of the sequenced isolate, ITS, βT and EF1-α gene regions were extracted from the assembly and a maximum likelihood phylogeny was constructed using the reference sequences of Van Wyk et al. (2010).

#### Results and discussion

**Fig. 2:**
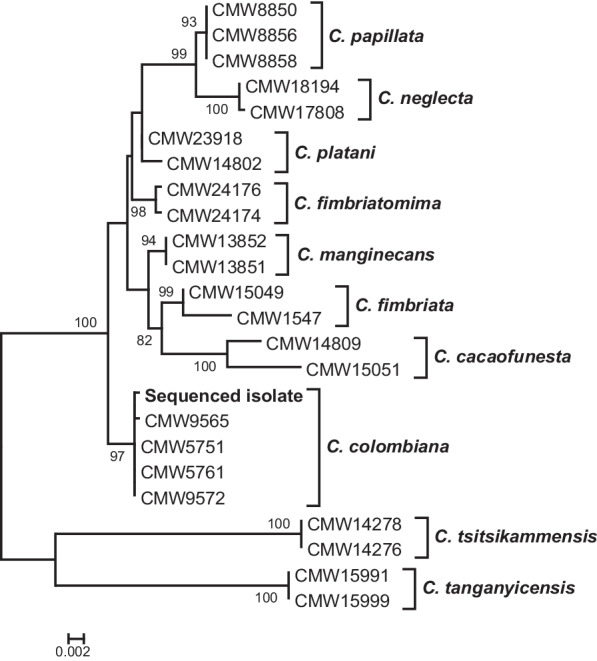
Phylogenetic tree generated from maximum likelihood analysis of the combined dataset ITS, βT and EF1-α gene regions. The sequences were extracted from the genome assembly of the sequenced isolate (bold type) and analysed together with reference sequences from Van Wyk et al. (2010). The *C. colombiana* (CMW5751) sequenced in this study resided in the same clade with original *C. colombiana* isolates described by Van Wyk et al. (2010)

A total of ~ 2,. million paired-end reads were obtained from Illumina paired-end sequencing, of which ~ 1.96 million pairs remained after trimming.* De novo* genome assembly with SPAdes followed by filtering steps resulted in a final assembly with 973 scaffolds and a N50 of 80.73 Kb. The genome assembly of *C. colombiana* is 31.19 Mb with a mean GC of 47.93%. The genome size of this species is in the same range as for other species of *Ceratocystis* sequenced to date; 27.31 Mb for *C. smalleyii* (Wingfield et al. 2018) and 32.15 Mb for *C. manginecans* (Fourie et al. 2020). Phylogenetic analysis using the extracted gene regions (ITS, βT and EF1-α) confirmed that the sequenced isolate was *C. colombiana* (Fig. 2), residing in the same clade with isolates originally described by Van Wyk et al. (2010). BUSCO analysis of the genome assembly using the sordariomycetes_odb10 dataset yielded a complete score of 94.6% and Augustus predicted 7358 protein coding genes encoded by the genome. The genome of *C. colombiana* from this study, together with those available for the genus and the larger *Ceratocystidaceae* will facilitate comparative genomics studies to understand evolutionary, pathogenicity and host adaptation of these economically important group of pathogens.

**Authors:** Tuan A. Duong^*^ and Brenda D. Wingfield.

^*^Contact: Tuan.duong@fabi.up.ac.za.

## IMA GENOME‑F 17C

### Long-read genome assembly of the maize grey leaf spot pathogen *Cercospora zeina* gives insight into its genomic architecture

#### Introduction

Grey leaf spot (GLS) is a foliar disease of maize that is widespread in sub-Saharan Africa (Meisel et al. 2009; Nsibo et al. 2021). The causal agents of GLS are two closely related species of *Mycosphaerellales* (*Dothideomycetes*), *Cercospora zeina* and *Cercospora zeae-maydis* (Crous et al. 2006). *Cercospora zeina* has a wide geographical distribution including the USA, Brazil, and China (Wang et al. 1998; Liu and Xu 2013; Neves et al. 2015). It is the causal agent of GLS in Africa, as DNA analysis confirmed that only *C. zeina* was present amongst 964 isolates from Kenya, Uganda, Zambia, Zimbabwe, and South Africa (Nsibo et al. 2021). The causal agent of GLS in Africa was named as *C. zeae-maydis* prior to splitting the GLS causal agent into two species, and the use of a reliable DNA diagnostic to distinguish it from *C. zeina* (Ward et al. 1999; Crous et al. 2006; Swart et al. 2017).

GLS caused by *C. zeina* results in great economic losses. Severe blighting of leaves in GLS susceptible maize genotypes reduces the supply of photosynthate during grain filling, with yield losses of 40–67% reported in South African field trials (Ward et al. 1999). A Zambian strain of *C. zeina* (CMW25467) has been used as an experimental model for studying infection of the maize host and the molecular biology of the pathogen (Meisel et al. 2009; Korsman et al. 2012; Swart et al. 2017; Meyer et al. 2017). A draft genome sequence of this strain was previously generated using short read data (Wingfield et al. 2017). However, this assembly was highly fragmented which limits its use in studying genomic architecture and evolutionary processes. To improve the genome assembly for the same strain (CMW25467) we employed long-read sequencing technology, since this approach can generate contiguous assemblies that span entire regions of repetitive DNA. The improved genome assembly will help to gain insight into the genomic architecture, proximity of coding genes to mobile elements, and serve as a foundation for syntenic comparisons with related phytopathogens.

#### Sequenced strain

**Zambia**: *Central region (Mkushi)*: isol. ex *Zea mays* (maize), March 2007, *F.J. Kloppers & B. Meisel* (CMW25467, MUCL 51677, CBS142763, PREM 61898—dried culture).

#### Nucleotide sequence accession number

This Whole Genome Shotgun project has been deposited at DDBJ/ENA/GenBank under the accession number MVDW00000000. The version described in this paper is version MVDW02000000. Biosample SAMN06067857; Bioproject PRJNA355276.

#### Materials and methods

*Cercospora zeina* strain CMW25467 (Meisel et al. 2009) was recovered from a glycerol stock stored at − 80 °C and maintained on V8 media. Greenhouse-grown maize B73 plants were inoculated with the strain. Once GLS lesions formed, single spore isolation was made to obtain a culture used for DNA extraction and sequencing. The gDNA was extracted using the CTAB protocol (Allen et al. 2006). The quality and quantity of the extracted DNA (3.2 μg) were assessed using 1% agarose gel electrophoresis and a Qubit 4 fluorometer.

PacBio sequencing was performed using one cell of a Single Molecule Real Time (SMRT) sequencer. The PacBio raw reads were assembled using the Hierarchical Genome Assembly Process (HGAP v4) included in SMRTLink v6 (Pacific Biosciences, CA) with default parameters. A polished assembly was generated using Arrow, which uses a statistical approach to generate a consensus sequence from the PacBio reads, as implemented in SMRTLink. The depth of coverage obtained by realignment of reads on the draft assembly was used to filter the assembly: we discarded contigs with a depth deviating by more than 1.5X from the average coverage across all contigs weighted by the contig length as in Plissonneau et al. (2016). To further improve the PacBio-based assembly, we used previously generated paired-end Illumina reads from the same *C. zeina* strain (Wingfield et al. 2017). We aligned the Illumina reads to the PacBio assembly using the Burrows-Wheeler Alignment (BWA) tool (Li and Durbin 2009), followed by one round of polishing with Pilon (Walker et al. 2014) with default settings. Statistics related to the assembly quality such as the N50 were measured with QUAST v4.4 (Gurevich et al. 2013). We addressed to which extent the PacBio genome assembly comprised complete chromosomes. To identify chromosome ends we used the programme Bowtie to identify the telomeric repeat CCCTAA in the final genome assembly (Langmead et al. 2009). This repeat motif has been identified in the telomeric regions of diverse organisms including plant pathogenic fungi (Fulnečková et al. 2013; King et al. 2015). We considered loci with at least ten times the repeat length (including one potential incorrect repeat) as putative sub-telomeric regions.

To annotate the genome, RNA-Seq reads from several *in planta* and *in vitro* sources (GSE99005, GSE94442, GSE90705, (Swart et al. 2017; Meyer et al. 2017)) were mapped onto the genome assembly using HISAT2 v2.1.0 (Kim et al. 2015) with the following parameters: --pen-noncansplice 18, --mp 6,0, --no-softclip, --max-intronlen 10,000, -t-reorder. The BAM file generated from HISAT was filtered to retain only concordant pair alignments and then used in the annotation of the genome using BRAKER v2.0.6 (Hoff et al. 2016) with the—fungus parameter. Genome assembly and annotation completeness were assessed with BUSCO v3.2.0 using the Ascomycota dataset (Simão et al. 2015).

OcculterCut v1.1 (Testa et al. 2016) was used to investigate whether the *C. zeina* genome was compartmentalized with respect to GC content. This method scans along the genome and detects adjacent segments of at least 1 kbp that have statistically significant GC content differences and are on either side of a position where the Jenson-Shannon divergence is maximized (Testa et al. 2016).

The species identity of the sequenced strain was verified by extraction of the translation elongation factor 1-alpha (TEF) and ITS sequences from the PacBio genome assembly, and phylogenetic comparison with related fungal species. Phylogenetic analysis was conducted using RAxML by applying the GTR + F0 + G10m model (Kozlov et al. 2019). The datasets for *TEF* and *ITS* nucleotide sequences were the same as those used to verify the species identity in the previous version of the *C. zeina* genome (Wingfield et al. 2017), with the exception that we used a more closely related outgroup (*Pseudocercospora oxalidis*) in this study.

#### Results and discussion

**Fig. 3:**
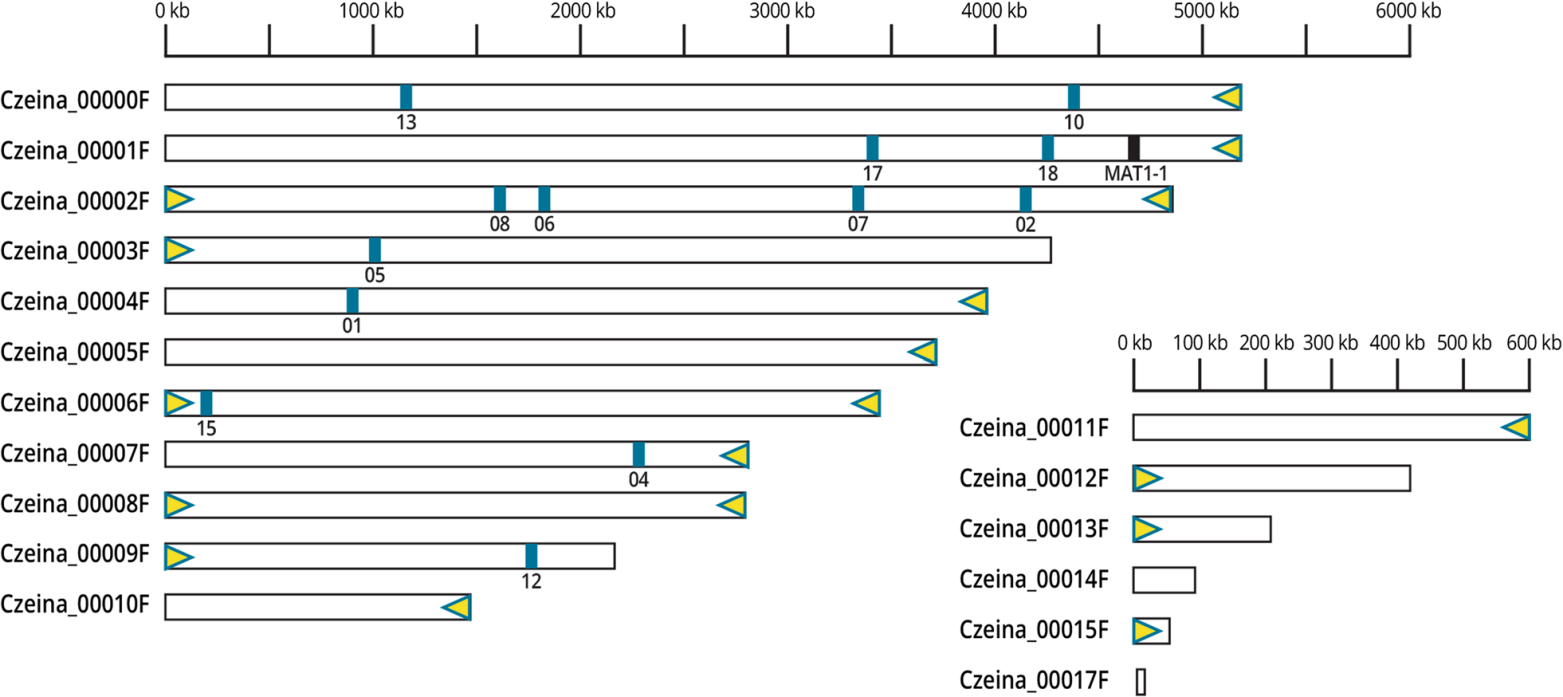
*Cercospora zeina* (CMW25467) PacBio genome assembly with positions of microsatellite markers and CCCTAA telomere repeats. The 17 nuclear contigs with protein coding genes are shown (Czeina_00000F-Czeina00017F). The five additional contigs 16F, 18F, 19F, 20F, 21F are not shown (sizes < 30 kbp). The telomere repeats are shown by yellow triangles. Contigs 02F, 06F, 08F had telomere repeats at both ends. The *MAT1-1* gene is located on contig 01F (black bar). Blue bars represent the locations of 13 microsatellite markers (with the CzSSR number below each bar) from (Muller et al. 2016). Contig 20F contained CzSSR11 (not shown). Different scale bars (kbp) are shown above contigs Czeina_00000F to Czeina_00010F; and contigs Czeina_00011F to Czeina_00017F

Genome assembly using HGAP v4 resulted in a 41 Mbp assembly consisting of 22 contigs of which 17 contigs had a mean coverage of 260X. Analysis focused on the 17 contigs since they corresponded to nuclear genome regions with protein-coding genes (Fig. 3; contig names shortened from Czeina_xxxxF to xxF in the text). The five contigs not included in the set of 17 had either a low (< 260X; contigs 16F, 18F, and 20F) or a very high mean coverage (> 1000X, contigs 19F and 21F). The assembled genome had an N50 of 4 Mbp and an L50 of 5. The longest contig was 5,172,692 bp (contig 00F) and the shortest contig was 29,391 bp (contig 17F). The telomeric repeated motif CCCTAA was present at both ends of three contigs (02F, 06F, 08F), indicating that these may represent complete chromosomes (Fig. 3). The telomeric repeated motif was also found at one end of twelve other contigs (Fig. 3).

The average GC content of the *C. zeina* genome was found to be 48%. We next investigated the variation in distribution of GC content along the genome assembly using the software OcculterCut (Testa et al. 2016). This analysis showed that approximately a third of the genome is made up of AT-rich segments (less than 41% GC content).

A total of 11,570 gene models were predicted from this new genome assembly, which is 1377 more than the 10,193 gene models predicted from a previously published Illumina-based genome assembly and annotation (Wingfield et al. 2017). Long-read sequencing with PacBio therefore revealed additional gene models that would not have been identified in a more fragmented assembly. The number of protein-coding genes predicted in the newly assembled *C. zeina* genome was similar to that of other Dothideomycetes determined by long-read sequencing  such as 10,528 to 12,386 in *Zymoseptoria* species (Feurtey et al. 2020), 11,257 in *Ascochyta rabiei* (Shah et al. 2020), and 14,186 in *Pyrenochaeta lycopersici* (Dal Molin et al. 2018). BUSCO analysis indicated that the assembly had a completeness score of 97.2% with 0.1% duplicated, 1.7% fragmented and 1.1% missing genes. The PacBio assembly reported here recovered five additional BUSCO genes that were not identified in the previous version of the assembly (Wingfield et al. 2017).

The five contigs that we had previously filtered out due to high or low coverage were also annotated to determine their gene content. BLASTN searches of the contigs 16F and 18F against the nr/nt database (NCBI) revealed sequences with similarity to annotated transposable element sequences of *C. zeae-maydis*. Contig 20F did not have any significant BLASTN match other than to a *C. zeina* microsatellite sequence (CzSSR11) (Fig. 3) (Muller et al. 2016). Contig 19F had sequence similarity to ribosomal RNA sequences including the 18S ribosomal RNA gene of various *Mycosphaerellales* including *Cercospora sojina*, *Zymoseptora tritici*, and *Cladosporium fulvum*. This suggested that contig 19F contains the ribosomal RNA cistron of *C. zeina*. The BLASTN search of contig 21F resulted in similarity to mitochondrial genome sequences of fungi, indicating that this may represent part of the *C. zeina* mitochondrial genome. None of the *in planta* nor *in vitro* RNA-Seq reads mapped to contigs 16F, 18F or 20F (data not shown). Finally, we also conducted an *ab initio* gene prediction of the sequences of the three contigs using AUGUSTUS, which did not identify additonal coding sequences. We conclude that the additional contigs are largely made up of repetitive sequences. However, further repeat element annotations are needed to verify this, and to identify the type of elements that they putatively encode.

**Fig. 4:**
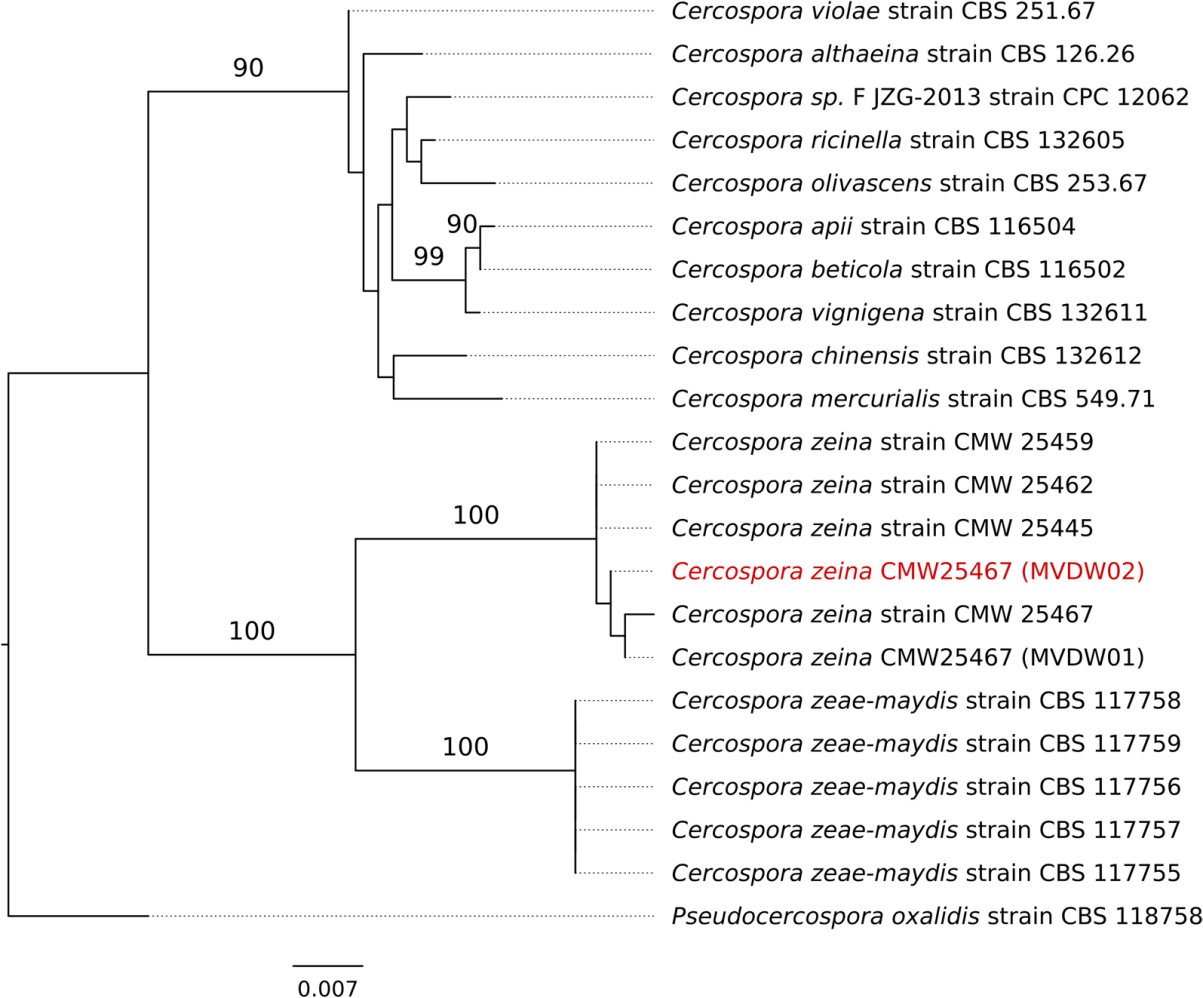
Phylogenetic tree generated to verify the phylogenetic relationship of the *Cercospora zeina* isolate sequenced in this study (MVDW02 indicated in red). Maximum likelihood analysis was performed on a concatenated dataset of translation elongation factor 1-alpha (*TEF1*) and *ITS* sequences, with percentage bootstrap (100) values shown. The outgroup was based on *Pseudocercospora oxalidis* sequences (*TEF1:* GU384467.1) and (*ITS*: GU269756.1). The branch length represents the mean number of expected substitutions per site. Fungal species and strain names are shown. Accession numbers for sequences can be found in Wingfield et al. 2017, where the previous short-read assembly (named MVDW01 in this Figure) was reported

The availability of the improved *C. zeina* assembly allowed us to locate the positions of microsatellites developed for population genetics studies (Muller et al. 2016) to determine whether linkage disequilibrium could affect their use in future. Previously, the sequences of the *C. zeina* CMW25467 alleles for 14 micro satellite markers had been determined by Sanger sequencing (NCBI accessions KP015832-42, KP015844, KP015846-47) (Muller et al. 2016). We located these on the *C. zeina* PacBio assembly using BLASTN, which showed that most of them were on different contigs or spaced more than 1 Mbp apart, except for CzSSR06 and CzSSR08 which were 200 kbp apart (Fig. 3). That most of these markers are not closely linked corroborates their usefulness where they have been used for population genetics studies of *C. zeina* isolates from East and southern Africa, and for future studies (Muller et al. 2016; Nsibo et al. 2019, 2021). The genome assembly from long-read sequencing determined in this study (MVDW02) was confirmed to be derived from *C. zeina*. This was shown by extraction of the sequences of elongation factor 1-alpha (*TEF1*) and *ITS* from the assembled genome sequence, followed by phylogenetic analysis with a dataset of these genes from related fungi. The phylogenetic tree showed that the source of the long-read assembly clustered with the same *C. zeina* strain (CMW25467) and other *C. zeina* strains, and that it was distinct from *C. zeae-maydis* and other *Cercospora* species, with strong bootstrap support (Fig. 4).

**Authors**: E.N.K. Kabwe, T. Welgemoed, T.A. Duong, L. Potgieter, A. Feurtey, D.L. Nsibo, E.H. Stukenbrock, and D.K. Berger^*^

^*^Contact: dave.berger@fabi.up.ac.za.

## IMA GENOME‑F 17D

### Draft genome sequence of *Elsinoë necatrix*: the causal agent of an emerging new and serious *Eucalyptus* foliar disease

#### Introduction

*Elsinoë* (*Elsinoaceae*) was introduced by Raciborski (1900) to accommodate a fungus that causes scab-like lesions on plant tissue. Members of these necrotrophic fungal pathogens are globally distributed and infecting approximately 70 hosts including important forestry, agricultural and horticultural crops as well as ornamental plants (Fan et al. 2017; Marin-Felix et al. 2019). Important diseases include the citrus scab pathogens *Elsinoë fawcettii* and *E. australis* (Chung 2011), the causal agent of grapevine spot anthracnose *E. ampelina* (Li et al. 2021), and the recently described *E. necatrix* that causes a devastating scab and shoot malformation disease on plantation-grown *Eucalyptus* (Pham et al. 2021).

Eucalyptus scab and shoot malformation was first reported in North Sumatra, Indonesia, in 2014 (Pham et al. 2021). The disease is characterized by black necrotic spots that first emerge on young leaves and petioles, which become scab‐like as the lesions age. Infected trees respond to infection by producing shoots with small leaves that commonly appear feathered. Severely affected *Eucalyptus* clones usually die after a number of successive infection cycles, generally over a period of two to three years. The disease has become prevalent across a large area of planted *Eucalyptus* in the region, resulting in significant damage (Pham et al. 2021).

Genome sequences are currently available for six economically important species of *Elsinoë.* These include *E. ampelina* (Haridas et al. 2020; Li et al. 2020), *E. arachidis* (Jiao et al. 2021; Su et al. 2022), *E. australis* (Shanmugam et al. 2020, Zhao et al. 2020), *E. batatas* (Zhang et al. 2022), *E. fawcettii* (Shanmugam et al. 2020; Jeffress et al. 2020) and *E. murrayae* (NCBI; https://www.ncbi.nlm.nih.gov). *Elsinoë necatrix* is amongst the more destructive of these pathogens and consequently a threat to commercial forestry in Asia and globally. The availability of its genome sequence will contribute to comparative genomics studies aimed at further understanding the biology of this important but relatively unknown group of pathogens.

#### Sequenced strain

**Indonesia:** *North Sumatra*: symptomatic leaf of *Eucalyptus* sp., 2020, *N.Q. Pham* (ex-holotype culture CMW56134 = CBS 147439; PREM 63209—holotype).

#### Sequence accession numbers

The genome sequence of *Elsinoë necatrix* (CMW56134) has been deposited in DDBJ/EMBL/GenBank databases under the accession number JANZYH000000000. The version described in this paper is JANZYH010000000.

#### Materials and methods

Genomic DNA was extracted from freeze-dried 5 day-old mycelium grown in malt yeast broth (2% malt extract, 0.5% yeast extract; Biolab, Midrand, South Africa) following the method described by Duong et al. (2013). Nanopore sequencing was conducted using the MinION sequencing device. The sequencing library was prepared using the Genomic DNA by Ligation (SQK-LSK109) protocol. The library was loaded on a MinION flow cell (R10.3) and sequencing was run for 48 h. Base calling was conducted using ONT Guppy base calling software v. 4.0.14 (https://community.nanoporetech.com). Porechop v. 0.2.1 (https://github.com/rrwick/Porechop) was used to remove adapters from the Nanopore reads. Illumina sequencing was carried out by Macrogen I (Seoul, South Korea), where the paired-end library was constructed and sequenced on NovaSeq 6000 Sequencing System to obtain 151 bp paired-end reads. The quality of the data obtained was assessed using the software FastQC v. 0.11.5 (Afgan et al. 2016). Trimmomatic v. 0.38 (Bolger et al. 2014) was used to remove poor quality data and the remaining Illumina adapters.

The genome was assembled with Nanopore data using Flye v. 2.7 (Kolmogorov et al. 2019) followed by polishing with raw nanopore reads using Racon v. 1.4.13 (Vaser et al. 2017) and Medaka v 1.0.3 (https://github.com/nanoporetech/medaka). To further improve the accuracy of the Nanopore assembly, two rounds of polishing with Illumina data were carried out using Pilon (Walker et al. 2014), where trimmed Illumina reads were aligned to the long-read contigs to generate a bam file used as input to polish the assembly. Protein coding gene models were annotated using the fungal version of GeneMark-ES (Ter-Hovhannisyan et al. 2008). The assembled genome completeness was evaluated using BUSCO v. 5.1.2 by using the Dothideomycetes dataset (Manni et al. 2021).

To validate the identity of the isolate, the internal transcribed spacer (ITS) region, the nuclear large subunit (LSU), part of the DNA-directed RNA polymerase II second largest subunit (*RPB2*) and the partial translation elongation factor 1-α gene (*TEF1*) regions were extracted from the assembly and analyzed together with references sequences of *E. necatrix* and other *Elsinoë* species obtained from GenBank. The sequence data set was aligned using the online version of MAFFT v. 7 (http://mafft.cbrc.jp/alignment/server/), (Katoh and Standley 2013). Phylogenetic analysis using maximum likelihood (ML) was performed with RaxML v. 8.2.4 on the CIPRES Science Gateway v. 3.3 (Stamatakis 2014) with GTR substitution model and 1,000 rapid bootstraps.

#### Results and discussion

**Fig. 5:**
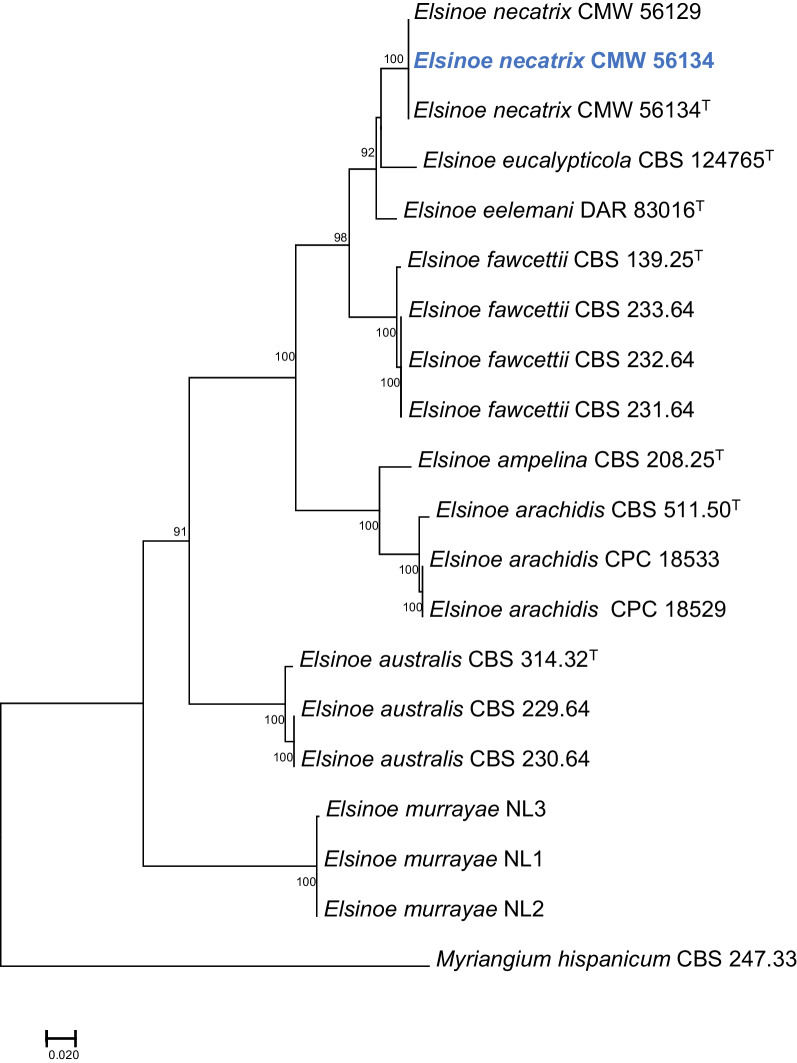
Maximum Likelihood tree based on ITS, LSU, *RPB2* and *TEF1* sequences for *Elsinoë* spp. Bootstrap values ≥ 90% for ML analyses are indicated at the nodes. Isolates representing ex-type material are marked with “T”. *Myriangium hispanicum* (CBS 247.33) represents the outgroup. The genome sequenced in this study, of which the sequences were extracted from the assembly, is indicated in blue

Nanopore sequencing generated 5.66 Gb data with read N50 value of 2.07 kb. Illumina sequencing generated 22.8 million paired-end 151 bp reads. The final assembly of *E. necatrix* (isolate CMW56134) consisted of 69 contigs, with the N50 of 0.73 Mb and L50 of 10. Phylogenetic analysis of four regions (ITS, LSU, *RPB2, TEF1*) confirmed the taxonomic identity of the isolate as *E. necatrix* (Fig. 5)*.* The assembled genome of *E. necatrix* was approximately 24.07 Mb with a GC content of 51.59%. The BUSCO completeness of the genome was estimated to be 93.42%: of the 3786 Dothideomycetes BUSCO groups searched, 53 BUSCO orthologs were reported to be fragmented, and 196 BUSCO groups were missing. GeneMark-ES predicted 9180 protein coding gene models in the assembled genome. The genome size and gene number for *E. necatrix* is relatively similar to that of other *Elsinoë* spp. (Table 1).

The draft genome sequence of *E. necatrix* generated here will facilitate future research regarding the biology and pathogenicity of this fungus. In particular, the genome sequence will be useful for developing molecular markers for population genetic studies to determine its origin and pathways of movement. This will have implications for the management of the disease and contribute towards a better understanding of the growing disease threats (Wingfield et al. 2013, 2015a, b) to *Eucalyptus* plantation forestry globally.

**Authors**: Pham NQ, Duong TA^*^, Wingfield BD, Barnes I, Durán A, and Wingfield MJ.

^*^Contact: Tuan.Duong@fabi.up.ac.za.

**Table 1:** Genome assembly features of *Elsinoe* spp.

Species	Strain	Accession number	Genome size (Mb)	Gene number	References
*Elsinoe ampelina*	YL-1	SMYM00000000	28.30	8057	Li et al. (2020)
	CECT 201119	JAAEIW000000000	28.27	10,207	Haridas et al. (2020)
*Elsinoe arachidis*	LNFT-H01	JAAPAX000000000	33.18	9174	Jiao et al. (2021)
	LY-HS-1	GWHBFXO00000000	32.44	9435	Su et al. (2022)
*Elsinoe australis*	Ea-1	SWCS00000000	23.79	9002	Shanmugam et al. (2020)
	NL1	NHZQ00000000	23.34	9223	Zhao et al. (2020)
*Elsinoe batatas*	CRI-CJ2	JAESVG000000000	26.49	9521	Zhang et al. (2022)
*Elsinoe fawcettii*	SM16-1	VAAB00000000	26.65	10,340	Shanmugam et al. (2020)
	DAR-70024	SWCR00000000	26.32	9930	Shanmugam et al. (2020)
	BRIP 53147a	SDJM00000000	26.01	10,080	Jeffress et al. (2020)
*Elsinoe murrayae*	CQ-2017a	NKHZ00000000	20.72	8281	NCBI
*Elsinoe necatrix*	CMW56134	JANZYH000000000	24.07	9180	This study

## IMA GENOME‑F 17E

### Short‑read genome assemblies and annotations of four *Pyrenophora teres* isolates collected from barley grass

#### Introduction

*Pyrenophora teres* (syn. *Drechslera teres*) is the causative fungus of net blotch disease in barley (*Hordeum vulgare*). Yield loss due to net blotch in susceptible barley cultivars can range from 10 to 70% (Jayasena et al. 2007; Wallwork et al. 2016) making *P. teres* one of the most important fungal pathogens of the barley industry world-wide. *Pyrenophora teres* exists as two forms; *Pyrenophora teres* f. *teres* (*Pitt*)  and *Pyrenophora teres* f. *maculata* (*Ptm*), causing net-form net blotch and spot-form net blotch in barley, respectively (Liu et al. 2011). Leaf symptoms due to *Pyrenophora teres* f. *teres* appear as dark-brown net-like transverse and longitudinal necrotic striations, while symptoms due to *P. teres* f. *maculata* develop as dark-brown circular to elliptic lesions on susceptible barley cultivars (Smedegård-Petersen 1971).

In addition to barley, *P. teres* can be found on weeds such as barley grass (*Hordeum leporinum,* subspecies of *H. murinum*) and other Gramineae crops such as wheat (*Triticum aestivum*) and oat (*Avena sativa*) (Shipton 1966; McLean et al. 2009; Khan and Boyd 1969). Even though *P. teres* has been reported to infect both cultivated and weed-like barley, host specificity of the pathogen is controversial (Linde and Smith 2019). Some studies reported that *P. teres* can infect host plant species belonging to the *Hordeum* family without being specific to any species in the family (Bakke 1912; Braverman 1960; Kenneth 1962), while other studies reported strict host specificity of *P. teres* to its host species (Khan 1973; Linde and Smith 2019). Either way, ancillary hosts like barley grass growing alongside cultivated barley can act as a source of inoculum for *P. teres* (MacNish 1964; Shipton 1966) and may play an important role in the evolution of this pathogen (Linde et al. 2016). Whole genome sequence data for *P. teres* isolates associated with weed-like barley hosts will contribute to better the understanding of the molecular mechanisms underlying its host association.

#### Sequenced strains

**Australia**: *Victoria*: Curyo, isolated from *Hordeum leporinum,* 2014, *J. Fanning* [*Ptm*14015] (BRIP 71574); Joel South, isolated from *Hordeum leporinum,* 2012, *M. Mclean* [*Ptt*12013] (BRIP 71573).*Queensland*: Yelarbon, isolated from *Hordeum leporinum*, 2010, *R. Fowler* HRS10128 (BRIP 71572). *Western Australia*: Mt Barker, isolated from *Hordeum leporinum*, 1995, *Rob Loughman* [SNB172] (BRIP 74832).

#### Nucleotide sequence accession number

This whole-genome shotgun project was deposited in the NCBI GenBank database under accession numbers JAMGBO000000000 (*Ptm*14015), JAMGBN000000000 (HRS10128), JAMGBM000000000 (*Ptt*12013) and JAMGBL000000000 (SNB172) [BioProject: PRJNA838266 and BioSamples: SAMN28416484 (*Ptm*14015), SAMN28416485 (HRS10128), SAMN28416486 (*Ptt*12013) and SAMN28416487 (SNB172)]. This paper describes the first versions of the four genomes.

#### Materials and methods

Genomic DNA of four barley grass isolates, *Ptm*14015, HRS10128, *Ptt*12013 and SNB172, was extracted from 14 to 20 day old fungal mycelium grown on half strength potato dextrose agar (PDA) medium [20 g/litre; Biolab Merck Darmstadt, Germany] using a Wizard^®^ Genomic DNA Purification kit (Promega, Sydney, Australia) as per the manufacturer’s protocol. The integrity of the DNA was assessed under ultraviolet light (Fusion FX, VILBER, Marne-la-Vallée, France) after agarose gel electrophoresis. DNA quantity and quality was measured with a NanoDropTC 2000/2000c and a NanoPhotometer P300 spectrophotometer (IMPLEN, Munich, Germany). The shotgun DNA libraries of *Ptm*14015, HRS10128 and *Ptt*12013 were constructed by Australian Genome Research Facility (AGRF, Melbourne) with 125 bp pair-end reads using TruSeq Nano library preparation kit. The shotgun DNA libraries of SNB172 was constructed with Nextera DNA XT library preparation kit by Macrogen (Seoul, South Korea) as per the Illumina short read protocol and sequenced with 150 bp pair-end reads on Illumina HiSeq 2000. The sequence quality of the pair-end reads of four genomes were examined by FastQC v0.11.8 (Andrews 2010). The adapter sequences of pair-end reads were trimmed by Trimmomatic v0.39 (Bolger et al. 2014). Sequences shorter than 40 bp and sequences with average phred score lower than 33 bp were also removed. The sequence quality of trimmed sequences was examined using FastQC. Trimmed sequences were used to perform* de novo* whole genome assembly with SPAdes v3.15.2 (Bankevich et al. 2012) by adjusting k-mer size from 20 to 40 bp. The quality and the completeness of all four genome assemblies were assessed using QUAST v5.0.2 (Gurevich et al. 2013) and BUSCO v.4.1.2 (Simão et al. 2015) with the fungi_odb10 database.

Repeat elements present in the genome assemblies were detected by RepeatModeler v1.0.11 (Smit and Hubley 2008), using Repbase v20.4 library (Bao et al. 2015) and masked using RepeatMasker v4.0.9 (Nishimura 2000). Gene prediction was conducted using BRAKER2 v.2.1.6 (Hoff et al. 2019) genome annotation pipeline, using the protein sequences of *P. teres* f. *teres* 0–1 (Ellwood et al. 2010) as protein evidences to train AUGUSTUS (Stanke et al. 2006).

**Fig. 6:**
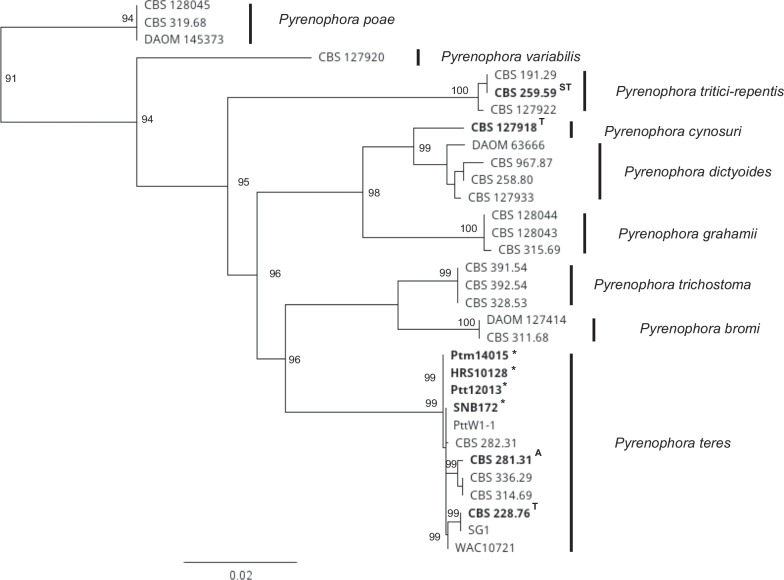
Maximum likelihood phylogram of *Pyrenophora* species constructed using RAxML v.8 (Stamatakis 2014) from the concatenated alignment of internal transcribed spacers and intervening 5.8S nrDNA (ITS), partial large subunit of the nrDNA (LSU), partial glyceraldehyde-3-phosphate dehydrogenase gene (*gapdh*), and partial DNA-directed RNA polymerase II second largest subunit gene (*rpb2*). The tip labels in bold and A, ST and T represent authentic, ex-syntype and ex-type strains (Marin-Felix et al. 2019), respectively, and asterisks denote isolates sequenced in the current study. Bootstrap support values > 90% are shown at nodes. The tree is rooted to *P. poae*

The verification of the sequenced isolates, *Ptm*14015, HRS10128, *Ptt*12013 and SNB172 as *Pyrenophora teres* was carried out by multi-locus phylogenetic analysis (Fig. 6) of four loci; internal transcribed spacers and the intervening 5.8S sequence of the nrDNA (ITS), partial large subunit of the nrDNA (LSU), partial glyceraldehyde-3-phosphate dehydrogenase gene (*gapdh*), and partial DNA-directed RNA polymerase II second largest subunit gene (*rpb2*). The sequences of the four loci of reference *Pyrenophora* strains were obtained from Marin-Felix et al. (2019) and Duong et al. (2021). The sequences of the four loci of the isolates in the current study were extracted from their respective genome assemblies. Multiple sequence alignment of each loci was conducted in MAFFT v.7.450 (Katoh and Standley 2013). Four multiple alignments from the four loci were then concatenated in Geneious Prime v.2021.2.2 (Kearse et al. 2012). A maximum likelihood phylogram of *Pyrenophora* species was derived from the concatenated alignment by RAxML v.8 (Stamatakis 2014) using the GTR substitution model with gamma-distribution rate variation for individual partitions and 1000 bootstraps. *Pyrenophora poae* was used as the outgroup for the phylogram (Duong et al. 2021).

#### Results and discussion

Illumina paired end (125 and 150 bp) sequencing of *Ptm*14015, HRS10128, *Ptt*12013 and SNB172 resulted in around 50 million reads each with ~ 100 × coverage of the whole genome. The final assemblies of *Ptm*14015, HRS10128, *Ptt*12013 and SNB172 genomes included 1295, 956, 1225 and 3384 contigs/scaffolds (≥ 1000bp) with N_50_ values of 111.57, 331.43, 109.55 and 348.92 kb, respectively. The CG contents of *Ptm*14015, HRS10128, *Ptt*12013 and SNB172 genomes were 46.71, 46.57, 46.59 and 47.92% and the largest contig size of each assembly was 596.68, 1508.41, 712.94 and 323.58 kb, respectively. The BUSCO completeness of the four assemblies ranged from 94.5 to 96.3% (Table 1). Out of 3786 total BUSCO genes searched, 3564, 3630, 3628 and 3604 BUSCO genes were found from the *Ptm*14015, HRS10128, *Ptt*12013 and SNB172 genomes, respectively (Table 1). The high completeness of these four genomes confirmed the high quality of the assemblies.

The compositions of the DNA transposons of the four genomes ranges from 1.16 to 2.66% of the total genome. The long interspersed nuclear element (LINE) composition of the genomes ranged from 0.28 to 1.16% and the long terminal repeat (LTR) retrotransposon ranged from 15.87 to 20.70% of the whole genome. The compositions of DNA transposons, LINEs and LTRs in the current study were similar to those previously reported for *P. teres* (Duong et al. 2021; Syme et al. 2018; Wyatt et al. 2020). The total number of protein-coding genes generated using BRAKER2 ranged from 11,038 to 11,314 among the four *P. teres* isolates (Table 1), which was greater than the 10,051 protein-coding genes reported for the *Ptt* and *Ptm* hybrid genome (Duong et al. 2021). These four genomes are the first published genomes for *P. teres* collected from barley grass (*Hordeum leporinum*) and will be highly valuable for future comparative studies of *Pyrenophora* species.

**Authors:** Buddhika Amarasinghe Dahanayaka, Barsha Poudel, Niloofar Vaghefi, Kiruba Shankari Arun-Chinnappa, and Anke Martin.

^*^Contact: Anke.Martin@usq.edu.au.

**Table 1:** Genome assembly statistics of the four *Pyrenophora teres* isolates from barley grass

	*Ptm*14015	HRS10128	*Ptt*12013	SNB172
*Assembly*				
Total assembly size (Mbp)	42.42	42.99	43.03	41.31
Number of contigs (≥ 1000 bp)	1295	956	1225	3384
N50 (kb)	111.57	331.43	109.55	348.92
Largest contigs (kb)	596.68	1508.41	712.94	323.58
GC-content (%)	46.71	46.57	46.59	47.92
*BUSCO analysis*				
Completeness	94.5	96.3	96.1	95.5
Complete and single-copy BUSCOs	3564	3630	3628	3604
Complete and duplicated BUSCOs	14	14	10	11
Fragmented BUSCOs	28	26	27	40
Missing BUSCOs	180	116	121	131
*Repeat annotation*				
DNA repeat elements (%)	1.16	1.24	1.52	2.66
LINEs (%)	0.28	3.20	0.31	1.16
LTR (%)	18.98	17.83	20.70	15.87
Unclassified (%)	6.26	4.95	5.28	6.88
Simple repeats (%)	0.73	0.66	0.73	0.66
Total gene annotation	11,069	11,038	11,068	11,314

## IMA GENOME‑F 17F

### Draft genome sequences for two isolates of the plant pathogen *Sclerotinia minor*

#### Introduction

*Sclerotinia minor* is a fungal pathogen that infects numerous plant hosts, including several crop species of economic importance (Melzer et al. 1997). It is closely related to the broad host-range pathogen *S. sclerotiorum* (Holst-Jensen et al. 1998), and these two species are often studied together due to their overlapping host ranges and similarities in both life cycles and mating systems (Subbarao 1998; Wu et al. 2008; Chitrampalam et al. 2013; Chitrampalam and Pryor 2015). Genome data for *S. minor* would be a useful resource for future research endeavours that could ultimately assist with managing this agricultural pathogen.

Limited genomic data is already available for *S. minor*. A high-quality reference genome sequence was produced for a single isolate isolated from lettuce in the Hubei province of China (Yang et al. 2022). This reference genome is 39 279 639 bp in length and consists of 24 scaffolds, with a GC content of 41.91%, which is similar to that of *S. sclerotiorum* (Amselem et al. 2011). Additionally, an unassembled genomic dataset generated by Illumina HiSeq 2500 sequencing for a single isolate of *S. minor,* isolate SsChi (Curtin University 2019), is available on the SRA database of the NCBI platform (Curtin University 2019). This isolate was obtained from common chicory (*Cichorium intybus*) in 1960, and the availability of a draft assembly for this isolate would be a useful addition to the current genomic resources for this pathogen.

In this genome announcement, the sequencing and assembly of a draft genome for a single isolate of *S. minor* is presented. This draft assembly is presented together with that of the isolate SsChi which is currently unassembled, and both these genomes were submitted to a public database. These genomes contribute to the available genomic resources for *S. minor* and other plant pathogens in the genus *Sclerotinia*.

#### Sequenced strains

**Italy:** isolated from *Lactuca sativa*, *G. Goidánich* (CBS 339.39, NBRC 6767, PREM 63312-dried culture).

#### Nucleotide sequence accession number

The Whole Genome Shotgun Project has been deposited at DDBJ/ENA/GenBank under the Accession Number JAMLGC000000000. The version described in this paper is JAMLGC010000000.

#### Materials and methods

*Sclerotinia minor* isolate CBS339.39 was obtained from the Westerdijk Fungal Biodiversity Institute and maintained on 2% PDA-ST media [20 g/L potato dextrose agar (Biolab, Merk, South Africa) supplemented with 150 mg/L streptomycin and 100 mg/L thiamine (Sigma, Steinheim, Germany)] at 25 °C for the duration of the study. For DNA isolation, the isolate was grown in a glass bottle containing 15 ml of 2% PDB medium (20 g/L potato dextrose broth) on an electronic shaker for 1–3 days at 25 °C. Mycelia were harvested by centrifugation in 50 mL centrifuge tubes at 4 °C for 10 min at 5000 rpm. The mycelia was freeze-dried before being subjected to genomic DNA extraction using a previously published method (Murray and Thompson 1980).

Genomic DNA was submitted to Macrogen (South Korea) for whole-genome sequencing. A TruSeq Nano library preparation was used for library construction with a 350 bp insert size. Sequencing was carried out on the NovaSeq platform, with target read lengths of 151 bp. The raw reads were imported as individual forward and reverse libraries into the Galaxy online platform (https://usegalaxy.org) (Afgan et al. 2018), and were subjected to a FastQC (version: 0.11.8) analysis to assess read quality. SPAdes (version: 3.12.0) was used for a *de novo* assembly of the draft genome sequence (Bankevich et al. 2012) using single-cell mode, k-mer options of 21, 33 and 55, and activating the careful correction option to minimize the number of short indels and mismatches. QUAST (version: 5.0.2) was used to assess the quality of the genome assembly and to determine the general statistics of the genomes (including genome size, GC content, N50, L50, number of contigs, largest contig size, and the average number of mismatches per 100 kbp) (Mikheenko et al. 2018). The BUSCO pipeline (version: 5.2.2) implemented in Galaxy was used to perform a quantitative assessment of the genome assembly completeness based on the fungi_odb10 dataset (Simão et al. 2015; Manni et al. 2021).

For a comparison, a second *S. minor* genome was also assembled. A single pair-end library (accession number SRX5407461) was obtained from the sequence read archive (SRA) database on NCBI (https://www.ncbi.nlm.nih.gov/sra) (Cochrane et al. 2011). This sequencing library was deposited by Curtin University in 2019 using the Illumina HiSeq 2500 sequencing platform (Curtin University 2019), and was derived from isolate SsChi that was isolated by F. Mujica from common chicory (*Cichorium intybus*) in 1960. The 33,971,728 reads of 125 bp average length were used in a stand-alone SPAdes assembly (version: 3.14) using “careful correction” and automated k-mer selection. The resulting assembly was again submitted to QUAST and BUSCO analyses to determine genome statistics and completeness, respectively.

To confirm the identity of the two *S. minor* draft genomes presented in this announcement, the partial small subunit ribosomal RNA gene, complete internal transcribed spacer 1 and 5.8S ribosomal RNA gene, and partial internal transcribed spacer 2 (ITS region) were extracted from the two assembled genomes (CBS 339.39 and SsChi) as well as from the LC41 reference genome (Yang et al. 2022). The sequences were added to a custom dataset of publicly available ITS sequences representing known *Sclerotinia* species, as well as a single sequence of *Botrytis cinerea* as outgroup. The dataset was aligned using MUSCLE (Edgar 2004) and curated with Gblocks using the Phylogeny.fr server (Castresana 2000; Dereeper et al. 2008). The curated dataset was used to construct a maximum likelihood tree in CLC Main Workbench (version 22.0.1, Qiagen). Additionally, an *in-silico* PCR amplification targeting the laccase 2 gene (*Lcc2*) was conducted in the CLC Main Workbench program using the “Find Binding Sites and Create Fragments” command. The PCR amplification made use of the laccase-specific primers developed as a diagnostic for *S. minor* (Abd-Elmagid et al. 2013), and was used to confirm the identity of isolate LC41 (Yang et al. 2022).

#### Results and discussion

Sequencing of the genomic DNA for *S. minor* strain CBS 339.39 on the Novaseq platform produced 35,898,334 reads with an average size of 151 bp. FastQC analysis confirmed that all reads were of high quality and were subsequently assembled into a draft genome of 37.9 Mb consisting of 16,992 contigs, 1908 of which were 500 bp or larger (Table 1). In comparison, the sequencing dataset of isolate SsChi obtained from the SRA archive was assembled into a genome of 36.8 Mb, consisting of 12,079 contigs of which 2091 were larger than 500 bp. This sequence was also submitted to the DDBJ/ENA/GenBank database under the Accession Number JAMLFZ000000000, with the specific version described here as JAMLFZ010000000. Despite the discrepancy in estimated genome size, both isolates had an identical GC content of 41.8% and a BUSCO completeness score of 99.5% (Table 1).

**Table 1:** The main metrics of the three available genome sequences of *Sclerotinia minor*

	CBS 339.39^a^	SsChi^b^	LC41^c^
*General genome statistics*			
Total genome length (bp)	37,906,295	36,885,191	39,279,639
Number of contigs (≥ 500 bp)	1908	2091	151 contigs / 24 scaffolds^d^
Total length (in contigs ≥ 500 bp)	35,755,541	35,701,198	39,279,639
Coverage	142x	115x	134–645x^e^
Largest contig size (bp)	202,369	150,738	4,022,415
GC (%)	41.82	41.80	41.91
N50	39,276	34,541	443,861
L50	276	319	7
Number of N’s per 100 kbp	32.27	36.89	60.86
*BUSCO completeness statistics*			
Overall completeness %	99.5	99.5	95.1
Total BUSCO terms	758	758	758
Single copy terms	754	754	718
Fragmented terms	1	1	3
Missing terms	3	3	34

^a^Isolate sequenced and assembled in this study

^b^Genome assembled from publicly available genomic data

^c^Previously published genome sequence (Yang et al. 2022)

^d^Assembled contigs were scaffolded using a published genome of *S. sclerotiorum* (Derbyshire et al. 2017)

^e^Coverage was reported for PacBio and MGISEQ-2000 sequencing, respectively

**Fig. 7:**
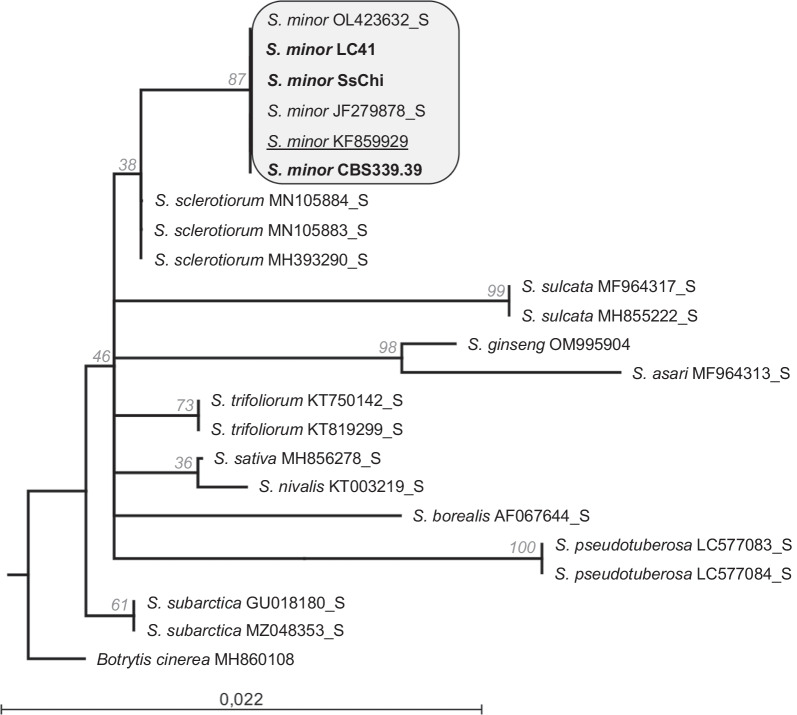
Maximum likelihood phylogeny of *Sclerotinia* species. The isolates used in the study (indicated in bold) are grouped with known isolates of *S. minor* (grey box), including a reference sequence for *S. minor* isolate CBS 339.39 (underlined)

Although the ITS region does not provide sufficient phylogenetic signal to distinguish *Sclerotinia* species (Holst-Jensen et al. 1998), the maximum likelihood tree produced from this region did produce a single clade containing all included isolates of *S. minor*, confirming the identity of the sequenced isolates (Fig. 7). The alignment of the ITS region for all the included *S. minor* isolates also showed 100% sequence identity (data not shown). The* in silico* PCR amplification predicted a 257 bp amplicon from both genome sequences using the *S*. *minor* diagnostic PCR primers, in line with the predicted amplicon size of 264 bp (Abd-Elmagid et al. 2013).

A previously published reference genome currently serves as the reference sequence for *S. minor* (Yang et al. 2022). This genome was from a hybrid assembly of long-read and short-read sequences that overall produces genome sequences of higher contiguity. The contigs were further scaffolded using the previously published genome sequence of the closely-related species *S. sclerotiorum* (Derbyshire et al. 2017) as reference. When compared to the two genome sequences presented here, the genome size of the LC41 reference strain is 1.3 Mb larger. This variation might have been caused by the choice of sequencing platform or assembly algorithm, or could reflect errors in the assembly and scaffolding process (Nagarajan and Pop 2013). Another interesting possibility is that these variations might reflect biological differences between the strains. The three isolates vary both in geographic origin (Italy, Chile and China) and host species (lettuce and chicory). Although some work has been done to unravel the genetic determinants of host specificity and population diversity in *S. sclerotiorum* (Aldrich-Wolfe et al. 2015; Derbyshire et al. 2022), this has not been echoed in *S. minor*. The availability of three genome sequences provides an exciting opportunity for future studies investigating the genomic basis of host specificity and pathogenicity for this important agricultural pathogen.

**Authors**: Chanel Thomas, Sikelela Buthelezi, Brenda D. Wingfield, and P. Markus Wilken.

^*^Contact: Markus.Wilken@fabi.up.ac.za.

## IMA GENOME‑F 17G

### Draft genome sequence of *Rosellinia necatrix* from avocado in South Africa

#### Introduction

*Rosellinia necatrix* is an ascomycete pathogen of plants in tropical, subtropical and temperate regions (Sivanesan and Holliday 1972; Petrini 1992). This fungus causes white root rot on many important crops, including apple, avocado, mango, and pear. *Rosellinia necatrix* invades the roots and crown which leads to the collapse of conducting vessels and eventually results in wilt and sudden death of infected plants (Pliego et al. 2009). Due to its resistance to chemical treatments, control and eradication of *R. necatrix* have become notoriously difficult (Pasini et al. 2016).

In South Africa, *R. necatrix* has been detected on apple and pear since the 1970s (Van der Merwe and Matthee 1974). In 2018, it was first reported to cause tree decline in avocado in the country (van den Berg et al. 2018). Since then, it has been spreading rapidly and is now present in all three major avocado-producing provinces of South Africa (Hartley et al. 2022). Nothing is known regarding the sources of *R. necatrix* introduction into South Africa nor its genetic diversity in the country. In this study, we report the genome sequence of a *R. necatrix* strain isolated from avocado in South Africa. This genomic resource, together with genome sequences of the species from other hosts and locations (Shimizu et al. 2018); https://www.ncbi.nlm.nih.gov/bioproject/727191), will facilitate comparative genomic studies and the development of genetic markers to study this important plant pathogen.

#### Sequenced strain

**South Africa**: *Limpopo*, isol. *Persea americana*, 2016, *J. Engelbrecht* (CMW50482, PREM 63335-dried culture).

#### Nucleotide sequence accession number

The Whole Genome Shotgun Project has been deposited at DDBJ/ENA/GenBank under the BioProject number PRJNA884201.

#### Materials and methods

Genomic DNA was extracted from a single hyphal culture of *R. necatrix* CMW50482 using the salt-based DNA extraction protocol of Aljanabi and Martinez (1997) with modifications (Duong et al. 2013). DNA was sent to Macrogen (Seoul, Korea) where a TruSeq PCR free library was constructed and sequenced on the HiSeq 2500 platform to generate paired-end reads of 251 bp. The sequence data were quality filtered and assembled in CLC Genomics Workbench v.22.0.1 (QIAGEN, Aarhus, Denmark). Quality filter parameters included a minimum quality limit of 0.02, removal of ambiguous nucleotides, removal of homopolymers at the 5′ and 3′ end when 9 out of 10 in a window are homopolymers and final reads had to be a minimum of 100 nucleotides. A *de novo* genome assembly was performed with default parameters, but with a bubble size of 100 and a word size of 40. Reads were mapped back to the contigs during assembly, which also updates the assembled contigs based on mapping information. The Genome Finishing Module v.22 was used to extend the contigs, using a minimum coverage cut-off of 10 reads and a maximum unaligned ends coverage cut-off of 30%. Contigs with a minimum length of 1 kb were selected to be included in the final assembly. Genome completeness was assessed based on the set of 1706 conserved Ascomycete single copy orthologs using BUSCO v.5.3.2 (Manni et al. 2021). AUGUSTUS v. 3.2.3 (Stanke et al. 2006) was used to annotate protein coding genes using the species model for *Fusarium graminearum*. To validate the identity of the isolate that was used for sequencing, the ITS gene region was extracted from the assembly and phylogenetic analysis was conducted with a reference dataset of *R. necatrix* and closely related species obtained from Hartley et al. (2022).

#### Results and discussion

**Fig. 8:**
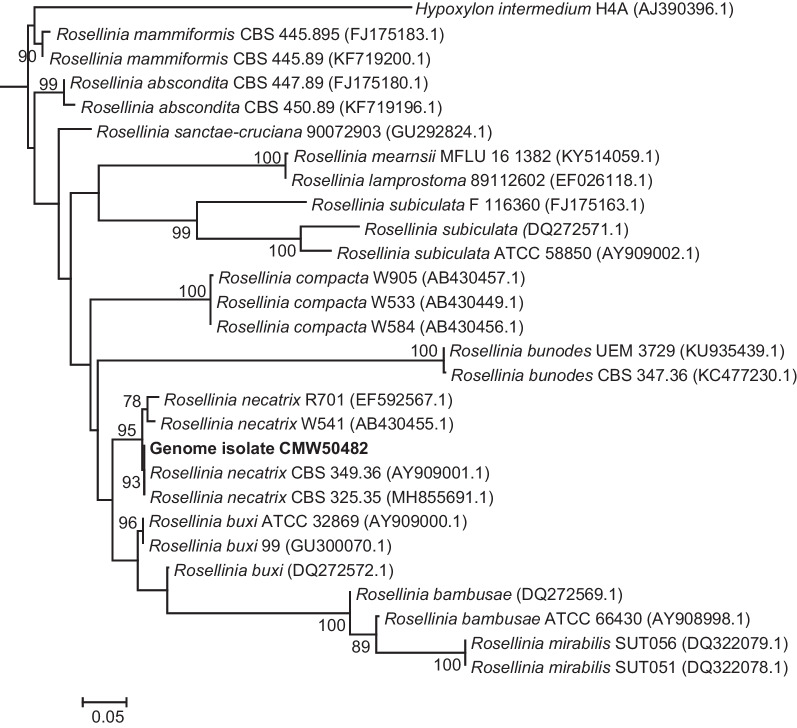
Identity confirmation of the *Rosellinia necatrix* isolate sequenced in this study (indicated in bold type). The maximum likelihood phylogeny was constructed from the ITS region using IQ-TREE v.2 (Minh et al. 2020). Bootstrap values larger than 75 are indicated at nodes. GenBank accession numbers are presented in the brackets

A total of 25.6 million paired-end reads were generated, of which 24.6 million paired reads with an average read length of 223 bp remained after trimming and quality filtering. The genome assembly resulted in 1362 contigs that were larger than 1000 bp with a maximum contig length of 560 kb and an N50 value of 124 kb, and thus the genome was sequenced with 65ʹ coverage. The assembled genome size was 48.79 Mb, with a GC content of 46.25%. Phylogenetic analysis using the ITS gene region extracted from the assembly confirmed the taxonomic identity of the sequenced isolate as *R. necatrix* (Fig. 8). The assembly size of *R. necatrix* in this study is larger than that of a previous sequenced isolate from pear in Japan (44 Mb: Shimizu et al. 2018), but in the same range with those from roses (48.7–49.2 Mb: https://www.ncbi.nlm.nih.gov/bioproject/727191), indicating the great variability in genomic composition of the species. BUSCO analysis indicated that the assembled genome had a 96.2% completeness score (0.1% duplicated and 3.8% missing). AUGUSTUS predicted a total of 10 714 protein-coding genes from the genome assembly. The genomic resource presented here represents the first *R. necatrix* genome from an isolate associated with avocado (*Persea americana*) and will contribute to a better understanding of the genetic mechanisms underlying pathogenicity and virulence of the species. This genome resource will also be useful for the development of molecular markers for population studies of this important species.

**Authors**: Juanita Engelbrecht, Arista Fourie, Jesse Hartley, Gerda Fourie, Noelani van den Berg.

^*^Contact: noelani.vandenberg@up.ac.za.

## Data Availability

The datasets generated during the current study are available in the NCBI repository, https://www.ncbi.nlm.nih.gov/bioproject/PRJNA355276/.
